# The effects of the first wave of COVID-19 restrictions on physical activity: a longitudinal study from “step into health” program in Qatar

**DOI:** 10.3389/fpubh.2024.1333546

**Published:** 2024-03-06

**Authors:** Abdulla S. Al-Mohannadi, Abdulaziz Farooq, Ahmad Salman, Amine Ghram, Sanaa T. Al-Harahsheh, Lina Majed, Suzan Sayegh, Marco Cardinale

**Affiliations:** ^1^World Innovation Summit for Health (WISH), Qatar Foundation, Doha, Qatar; ^2^Aspetar, Orthopaedic and Sports Medicine Hospital, Research and Scientific Support, Doha, Qatar; ^3^Department of Public Health Practice, Kuwait University, Safat, Kuwait; ^4^Department of Physical Education, College of Education, Qatar University, Doha, Qatar; ^5^Department of Cardiac Rehabilitation, Heart Hospital, Hamad Medical Corporation, Doha, Qatar; ^6^Research Laboratory "Heart Failure, LR12SP09", Hospital Farhat HACHED of Sousse, Sousse, Tunisia; ^7^Healthy Living for Pandemic Event Protection (Hl-Pivot) Network, Chicago, IL, United States; ^8^College of Health and Life Sciences, Qatar Foundation, Hamad Bin Khalifa University, Doha, Qatar; ^9^Modern University for Business and Science, Beirut, Lebanon; ^10^Department of Targeted Intervention, University College London, London, United Kingdom; ^11^Sport, Exercise and Rehabilitation, Northumbria University, Newcastle upon Tyne, United Kingdom

**Keywords:** physical activity, pedometer, COVID-19, pandemic, lockdown, longitudinal study

## Abstract

**Introduction:**

The COVID-19 pandemic led to restrictions that prevented physical activity in public places. This study sought to conduct a comprehensive longitudinal analysis of how lockdown policies in an Arabian Gulf country influenced the patterns of physical activity during first wave.

**Methods:**

In a longitudinal study design, members of the ongoing “Step into health” community-based health promotion program who provided valid pedometer data from January to August 2020, covering pre, during and post-covid first wave period met the inclusion criteria.

**Results:**

420 (76.7% men, 13.8% ≤40 years) were included in the study. Overall, significant decline in daily step counts was recorded (−1,130 ± SE302) after the implementation of lockdown policies (*p* < 0.001). When the restrictions were removed, the steps per day were still lower compared to pre-covid for men (−910 ± SE610, *p* = 0.017) and among individuals with normal BMI (−1,304 ± SE409, *p* = 0.004). The lockdown in Qatar did not significantly affect women and individuals with obesity who already had lower daily steps pre-covid.

**Discussion:**

The present study confirms immediate decline in daily steps imposed indirectly through the COVID-19 lockdown measures. Participants with higher physical activity levels pre-covid experienced significant decline in step count during and even after restrictions were uplifted.

## 1 Introduction

At the onset of the first wave of the coronavirus disease (COVID-19) in the State of Qatar, the Ministry of Public Health implemented a nationwide lockdown and restrictive measures that included social distancing, quarantine and self-isolation. Although lockdowns were essential to limit the spread of the disease, they brought about important changes in lifestyles and behaviors that had undeniable consequences on mental (1) and physical health (2). With the closure or limited access to work places, educational institutions, outdoor spaces, or fitness/sports centers, people spent most of their time confined at home, either working or engaging in leisure screen-based activities (3). In addition, many adults were also presented with increased household and childcare commitments. As a result, a general decline in physical activity was reported in most studies in different parts of the world (4) as well as an increase in sedentary behaviors during COVID-19 lockdown (5, 6).

The World Health Organization (WHO) recommends adults to engage in a minimum of 150 min of moderate-intensity or 75 min of vigorous-intensity physical activity per week for substantial health benefits (7). Engaging in regular physical activity is crucial for maintaining good health, preventing chronic non-communicable diseases, and improving mental health and well-being (8). Being active has also been linked to lowering the detrimental impact of COVID-19 measures on health as well as reducing the gravity of the infection (9, 10).

As the COVID-19 pandemic continued, the WHO and other international organizations issued specific physical activity guidelines for times of lockdown (e.g., home-based exercises), urging people to remain active and break up sedentary behavior (11). In addition, the most reported barrier to physical activity (i.e., lack of time, (12) was no longer a concern for some people for which an increase in physical activity was observed during lockdown (13, 14)). However, despite the fact that some were presented with new opportunities to remain or become physically active, a general decline in all intensity levels of physical activity (15) and increase in sedentary behaviors were still observed for various populations including children and medical patients (16).

Four studies from the Arabian Peninsula also indicated such declines in physical activity. For instance, 52% of the 2,255 adult participants from Saudi Arabia reported a decrease in their physical activity levels, which was significantly associated with gains in weight (17). In UAE, (18) revealed that 38.5% of the 1,012 adult participants did not engage in physical activity and 36.2% of them spent over 5 hours per day on screen-time activities for entertainment. Additionally, a third of adults living in the State of Kuwait reported engaging in less than 30 min of physical activity or exercise per week during the COVID-19 lockdown (19). Finally, a study conducted in Qatar found that the COVID-19 quarantine also negatively impacted physical activity levels, resulting in a decrease in physical activity (especially moderate-intensity), increase in sitting time, and fewer walking days of at least 10 min per week (20). However, to our knowledge, all studies from the Gulf Peninsula region used subjective measures (i.e., self-reported questionnaires and surveys), lacking objective assessment of physical activity patterns.

In response to the challenges posed by the COVID-19 pandemic, many countries have closely monitored national public health programs. The 10,000 Steps Australia program that included more than 400,000 participants showed that the detrimental effects of lockdown on step count were consistent across age groups and genders (21) and disappeared after the ease of restrictions. Another longitudinal study from Japan found decreases in average step count during the lockdown period, mostly affecting women and non-older people (22). The Step Into Health (SIH) program in Qatar is one such initiative, engaging the community to adopt a more active lifestyle by promoting walking (23). The availability of pedometer data from the Qatar’s SIH program offers a rare opportunity to gain an objective understanding of the impacts of the COVID-19 pandemic on physical activity. Therefore, the purposes of the present study were to: objectively assess changes in daily steps during the first wave of the COVID-19 pandemic and lockdown in Qatar and identify the population characteristics associated with these changes. In Qatar, half of the population do not engage in regular physical activity, while 82% of middle-aged women do not engage in any form of physical activity (24). However, it is important to mention that participants in such studies are usually considered “diligent” as formulated by (22) or motivated to use pedometers on a daily basis and regularly upload their records. Therefore, the analysis of a specific “health-conscious” part of the population with daily step counts higher than reported averages in the general population (21, 22) would provide unique insights into the impact of the pandemic policies on a relatively active group. The value of the findings will reside in the ability to use the information to inform policy in case of future pandemics and/or limitations in public sports activities.

## 2 Materials and methods

### 2.1 Study design and population

This is a longitudinal study which aims to assess the changes in daily steps during the first wave of the COVID-19 pandemic and lockdown in Qatar and identify the population characteristics associated with these changes. SIH, a community-based program, was launched in 2012 to promote physical activity among the residents of Qatar (23). The program was publicized nationwide through outreach advertisement campaigns within different settings (workplaces, campuses, and malls). This program encourages participants to increase their overall steps up to 10,000 steps per day or more. Upon registration, participants were provided with a free of charge pocket-sized Omron HJ-324 U pedometer (Omron Healthcare, Co., Ltd., Kyoto, Japan) linked to a web-database that records their activity levels. During the study period of interest, there were 1,409 registered pedometer users in the program that were active.

### 2.2 Study measures and participants

Demographic and anthropometric data were extracted from the database, including age, gender, nationality, and Body Mass Index (BMI) based on self-reported height and weight. As for physical activity assessment, daily and aerobic step counts of pedometer records from the database were extracted for the studied period. Participants were included in this study if they provided daily pedometer data between 1st of January and 30th August 2020, (marking pre, during and post COVID-19 wave 1 outbreak phase). Individuals who did not upload physical activity data were excluded. To ensure valid wear time, only observations with step counts ranging from 500 to 60,000 were included (23).

### 2.3 Ethical considerations

Participants were asked to sign a disclaimer upon registration to the SIH program, agreeing to the use of their data for program evaluation and research. The data was anonymized prior to analysis and personal information was treated with confidentiality. The study adhered to ethical guidelines and was approved by the Institutional Review Board of Aspire Zone Foundation (E202104021).

### 2.4 Timeline of wave 1 of COVID-19

On February 29, 2020, the first case of COVID-19 involving a Qatari male returning from Iran was confirmed. Lockdowns were implemented on March 12, 2020, leading to the closure of various public venues like theaters, children play areas, gyms etc. In the initial phase of lifting of restrictions, starting from June 15, mosques opened with precautions, along with 40% capacity granted to selected private healthcare facilities. Malls partially resumed operations while maintaining limited access, and outdoor sports were permitted within restricted park areas. Workplaces were allowed to function with 20% capacity, adhering strictly to health protocols. From July 1, the second phase commenced, permitting small gatherings of up to 10 people. Further openings for mosques were initiated, alongside a 60% operational capacity for private health clinics. Parks, Corniche, and beaches were made accessible, with malls operating under restricted hours and capacity. Additionally, 50% workplace capacity was permitted with stringent health precautions. Moving into the third phase starting from August 1, medium-scale gatherings of up to 40 people were allowed. Mosques were open for Friday prayers, and private health clinics were permitted to function at 80% capacity. Professional sports training was allowed in open spaces and large halls, with a limit of five people. Health clubs, gyms, pools, beauty and massage parlors, and barber shops operated at 50% capacity. All malls were open for full hours, and restaurants began operating with limited capacity, gradually increasing over time.

### 2.5 Data analysis

Data were analyzed using SPSS (Statistical Package for Social Sciences) v21.0. Descriptive statistics, including means, standard deviations were used to represent daily step count. Participants age were grouped into two categories (i.e., age ≤ 40/>40 years) based on distinct health and fitness goals and varying prevalence of age-related chronic conditions from earlier reports in Qatar (25). The participants were also categorized into four regional groups according to the World Health Organization classified regions (WHO). BMI Status was calculated and categorized into three categories (i.e., ≤25 normal, >25 to <30 overweight, and ≥ 30 Obese). To study the changes in daily step count, a moving average of 7 days was used on a time series graph to visualize the changes by age, gender, BMI status and region. To isolate the impact of COVID-19-related lockdowns from climatic conditions on daily physical activity, we conducted a comparative analysis of daily step counts during equivalent months or periods in the years 2019 (as a reference) and 2020. Linear mixed models were used to assess the changes in steps per day during and after COVID-19 lockdown, compared to baseline assessment, based on independent factors such as sex, age, and obesity status. All factors and their interaction with factor time were included in the linear mixed model. By adding subject id as a random effect with a random intercept we accounted for individual-level variation and within-subject correlation. Unstructured covariance structure was found to be the best fit. Region could not be included in the above model due to small sample size of some groups especially the participants from the African continent. Separately a linear mixed model was designed that included time and region interactions.

## 3 Results

Four hundred and twenty individuals (average age 50.0 ± 9.4 years) met the inclusion criteria in this study. A significant majority of the participants, accounting for 86.2% of the total sample were older than 40 years.

The study comprised predominantly male participants, (76.7%). Regarding BMI categorization, a substantial portion of the sample (46.0%) fell within the overweight category and 21.4% were obese. Geographically, the majority of the participants hailed from the Southeast Asian region (60.2%), followed by the Middle Eastern region at 31.9% (Table 1).

**Table 1:** Characteristics of the participants.

Variables		Frequency	Percentage
Age group			
	≤40 years	58	13.8%
	>40 years	362	86.2%
Sex			
	Females	98	23.3%
	Males	322	76.7%
BMI group			
	≤25 kg/m^2^	137	32.6%
	25–30 kg/m^2^	193	46.0%
	>30 kg/m^2^	90	21.4%
Region			
	African	7	1.7%
	Middle Eastern	134	31.9%
	Southeast Asian	253	60.2%
	Western	26	6.2%

Table 2 presents the changes in the steps per day during and after the COVID-19 lockdown, compared to the baseline step count according to the participant characteristics.

**Table 2:** Linear mixed model: steps per day (mean ± SE)* and changes in steps per day during and after COVID-19 lockdown compared to pre-covid.

	Pre-covid	During COVID lockdown	Post lockdown	During vs. pre (Percentage of change)	*p-*value	Post vs. pre (Percentage of change)	*p-*value
Overall	7,969 ± 376	6,915 ± 421	7,275 ± 371	−1,054 ± 309 (−13.2%)	0.002	−694 ± 293 (−8.7%)	0.054
Sex							
Female	7,686 ± 591	6,922 ± 651	7,320 ± 570	−763 ± 442 (−9.9%)	0.235	−366 ± 413 (−4.8%)	0.758
Male	8,253 ± 388	6,908 ± 441	7,230 ± 393	−1,345 ± 319 (−16.3%)	<0.001	−1,023 ± 299 (−12.4%)	0.002
Age group							
≤40 years	7,472 ± 546	6,000 ± 625	6,572 ± 590	−1,472 ± 530 (−19.7%)	0.017	−900 ± 509 (−12.0%)	0.215
>40 years	8,466 ± 348	7,830 ± 386	7,978 ± 333	−636 ± 250 (−7.5%)	0.034	−489 ± 231 (−4.8%)	0.102
BMI Status							
≤25 kg/m2	9,313 ± 543	7,858 ± 599	7,826 ± 525	−1,454 ± 407 (−15.6%)	0.001	−1,487 ± 377 (−16.0%)	<0.001
>25 to <30 kg/m2	7,620 ± 476	6,537 ± 533	7,199 ± 469	−1,083 ± 359 (−14.2%)	0.008	−421 ± 335 (−5.5%)	0.507
≥30 kg/m2	6,976 ± 629	6,350 ± 709	6,800 ± 619	−626 ± 480 (−9.0%)	0.476	−176 ± 450 (−2.5%)	0.972

**Figure 1:**
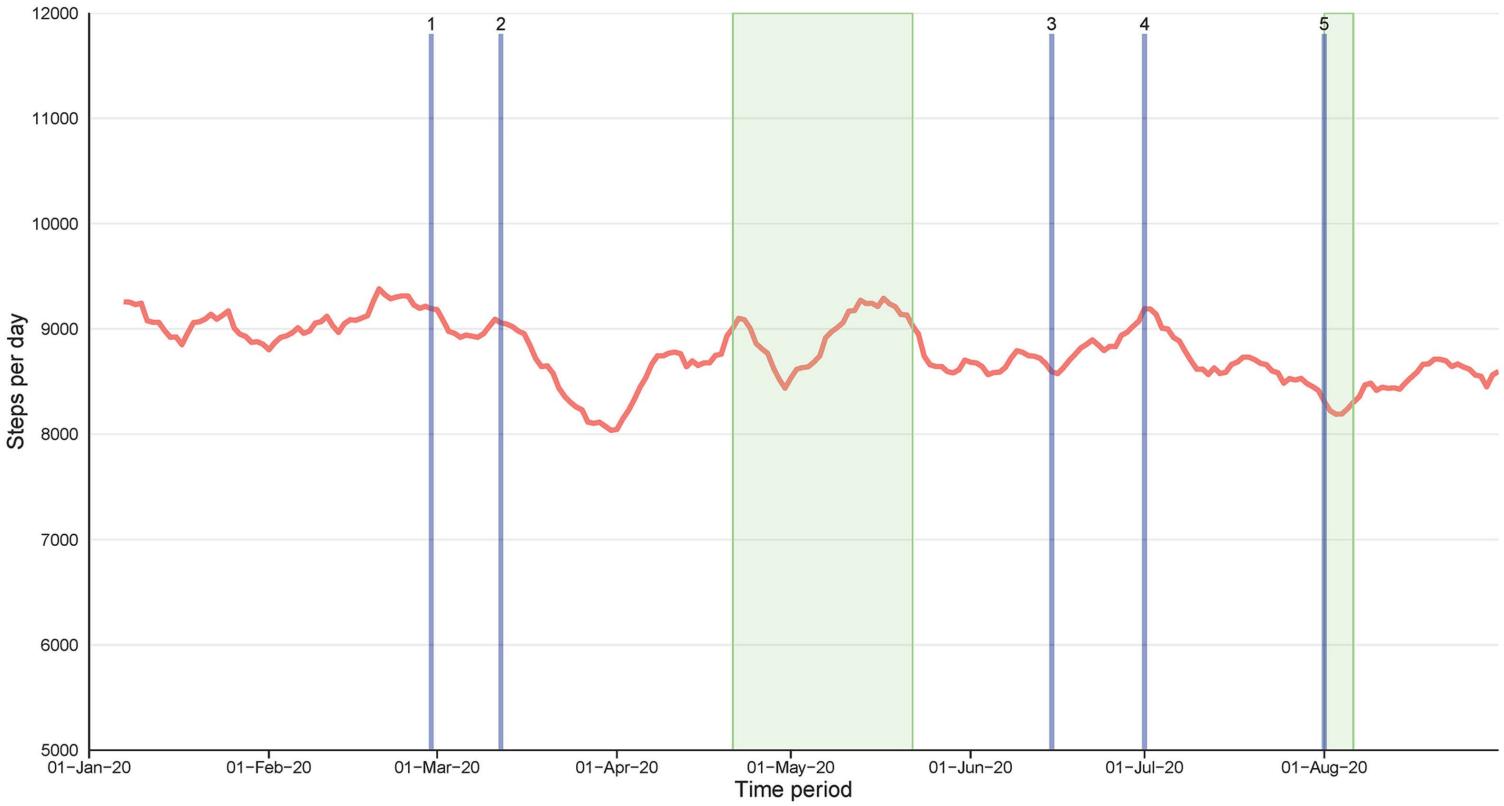
The moving average (7 d) steps per day among the participants from January 2020 to 31 August 2020. The wide, green-shaded area represents the month of Ramadan (23 April 2020–23 May 2020) and the narrow, green-shaded area represents Eid al-Adha observation (30 July 2020–6 August 2020). For all figures, the description of the timepoints is as follows: (1) First COVID-19 case in Qatar; (2) Qatar imposes lockdowns; Gradual lifting of lockdown (3) Phase 1; (4) Phase 2; (5) Phase 3.

After lockdown restrictions were implemented, the step count steeply fell and average steps thereafter increased even before the lifting of restrictions (15 Jun 2020) throughout the lockdown until the beginning of Ramadan, the month of fasting (see Figure 1).

**Figure 2:**
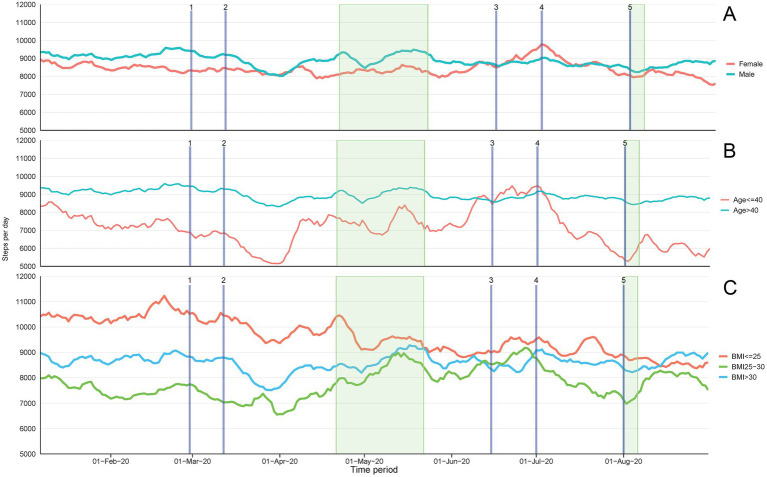
The moving average (7 d) steps per day among the participants, based on sex, age group, BMI status, from January 2020 to 31 August 2020. The wide, green-shaded area represents the month of Ramadan (23 April 2020–23 May 2020) and the narrow, green-shaded area represents Eid al-Adha observation (30 July 2020–6 August 2020). The description of the timepoints is as follows: (1) First COVID-19 case in Qatar; (2) Qatar imposes lockdowns; Gradual lifting of lockdown (3) Phase 1; (4) Phase 2; (5) Phase 3.

Compared to the pre-pandemic step count, during the lockdown step count was reduced by −1,054 ± 309 steps (13.2%, *p* = 0.001). By contrast, the after-pandemic step count was still lower 694 ± 293 steps, but this difference was not statistically significant (*p* = 0.054) (Table 2). Before the pandemic, men were generally more active than women. At the beginning of the lockdown, a decrease in steps was evident fell in women (−763 ± 442 steps) [albeit not reaching statistical significance with *p* = 0.235] but was larger and statistically significant in men (−1,345 ± 319steps [16.3%, *p* < 0.001]). Compared to the pre-pandemic step count, the after-pandemic step count in men was still lower by −1,023 ± 299 steps (12.4%, *p* = 0.056) and in women but not statistically significant (4.8%, *p* = 0.974) (See Figure 2A). An unexpected finding was that the younger individuals in the SIH cohort (i.e., <40 years old) were less active than older individuals (i.e., ≥40 years old) before, during, and after the pandemic (See Figure 2B). The decrease in step count during the pandemic versus before the pandemic in the <40 years age group was (−1,472 ± 530, *p* = 0.017) and in the ≥40 years age group was (−636 ± 250, *p* = 0.034).

Significant differences were also identified between the activities of individuals who were classified as normal weight (BMI: 18.5–24.9), overweight (BMI: ≥25 to <30), and obese (BMI: ≥30). The step count pre-covid was lower in individuals with higher BMI (see Figure 2C). During the pandemic, compared to before the pandemic, the step count decreased significantly by −1,454 ± 407 steps in normal weight people and by −1,083 ± 350 steps in overweight people (*p* = 0.001 and *p* = 0.008, respectively). In the obese group, the step count decreased by −626 ± 480 steps, but this difference was not significant (*p* = 0.476). The after-pandemic step count in the normal weight group was still lower post pandemic restrictions (−1,487 ± 377 *p* < 0.001) but remained similar in other groups as clustered by BMI status.

During the lockdown and during the lifting of restrictions, the activity level of the Middle Eastern individuals and the Southeast Asian individuals did not change substantially. By contrast, the activity level in Western individuals increased during the lockdown, however, it decreased with each phase of the lifting of COVID-19 restrictions. Due to small group sizes of ethnicity, region could not be included as a factor in the multivariate linear mixed models shown in Table 2. In a univariate analysis, only looking at the effect of time period on step count by regions, it is shown that average step count during the pandemic was significantly reduced in Southeast Asian group (−1,247 ± 230, *p* < 0.001) and this remained significantly lower during post pandemic restrictions (−876 ± 310, *p* = 0.015). The average steps per day among Western participants remained above 10,000 steps throughout the lockdown period.

By comparing physical activity time series data during the 2020 study period with physical activity of 2019 as a reference we observed distinctive declines in step count during lockdown period (data not shown). This approach allowed us to ensure that observed changes were primarily attributed to the effects of lockdown measures rather than seasonal variations in weather.

## 4 Discussion

The present study aimed to assess the impact of the first wave of COVID-19 lockdown and fluctuations in daily step counts among citizens and residents of Qatar. It is the first of its kind to use objective pedometer data to measure changes in physical activity during and after the first wave of the pandemic in Qatar. The main result of the study revealed a significant overall decline (i.e., −13.2%) in daily steps during the lockdown period and a recovery with the lifting of the restrictions, which is consistent with previous studies in other parts of the world (21, 22). Although the average decline in step counts was approximately of 1,000–1,500 steps per day, walking an extra 1,000 step per day can reduce risk of cardio-vascular disease and all-cause mortality by 5–21 and 6–36%, respectively (26). Therefore, a relatively small reduction can have marked effects, in particular in individuals which are not particularly active.

Interestingly, a differential impact of the lockdown was observed based on characteristics of subgroups. In fact, step counts of women and individuals with obesity (i.e., BMI ≥ 30) did not vary significantly after the lockdown or after the lifting of restrictions. Conversely, step counts of men and those with normal weight-status (BMI ≤ 25) significantly decreased during that period and remained lower than baseline consistently across age groups. This first finding might suggest that groups who were the most impacted by the restrictive measures were those who presented higher step counts at baseline. Indeed, many studies demonstrated a larger effect of lockdown on people who were initially more active, including men (13, 27). Conversely, while all age groups were significantly affected by the lockdown, the older group (i.e., > 40 years) who was initially more active than the younger one (i.e., < 40 years) was less severely affected by the lockdown and the lifting of restrictions. Although this finding might seem counter-intuitive for this “more vulnerable” group, it aligns with what was found in an Australian study (21). Authors argued that the older individuals have a higher emotional stability and lower reliance on team-based activities, use of gyms and sporting facilities, which allowed them to maintain their step count. It is also assumed that the older group tended to engage is leisure-time activities that would include walking and was more health conscious to potential health risks of reducing their physical activity level (28). These findings have important implications for informing future public health policies and interventions during times of crisis that should not only focus on the most vulnerable and sedentary subgroups (i.e., older or obese individuals or women), but also on individuals with high activity levels which experience the larges change in activity behaviors.

These results are somehow similar in what was reported in a British cohort where most of the participants <35 years of age did not report sufficient physical activity during the social distancing policy implementation phases (27). Two other studies reported a much larger reduction in PA in males when compared to females in Greece and in the USA. The significant decrease in physical activity observed by these authors (13, 29) in men but not in women during the COVID-19 pandemic may be attributed to various factors. Indeed, understanding gender differences in choices and motivations toward physical activities might offer a good insight. As compared to women, men tend to engage in more outdoor, team-based, high-intensity and competitive physical activities and sports which were more restricted during the pandemic due to social distancing guidelines (13, 30). Women, on the contrary, tend to engage in physical activities for reasons that are more related to weight management or physical appearance as found in a study from Greece (13). Furthermore, men tend to have more demanding work schedules and may have faced additional work-related stressors during the pandemic, leading to decreased physical activity levels (31). Additionally, lockdown and social distancing policies might have determined increased responsibilities for men at home, such as caring for children or older people, which could have limited their time for physical activity observed in our and other studies.

The time-series analyses were able to depict further important fluctuations with the different key events and variations between behaviors of sub-groups during this eight-month period in Qatar. Generally, after the steep decline in step counts due to lockdown, step counts started increasing only a few weeks later and before the start of the first phase of lifting of restrictions (3) (Figure 1). Figure 2 show higher baseline step count for men, normal weight individuals, older and western sub-groups with differences remaining consistent during the full period. However, large variations are seen in men (*vs* women) and the younger group (*vs* older) / [obese group (*vs* normal weight) and western group (*vs* other regions)] at different stages that relate to the lifting of restrictions, Ramadan or holidays (Eid Al-Adha) potential indicating efforts to remain active or regain activity level. The higher fluctuations are also indicative of more responsiveness or sensitivity to changes in the social and environmental context and therefore a higher vulnerability to key events happening.

From our previous analysis in this settings (23), we are aware the climate does play a role in impacting daily physical activity behavior, hence by looking at daily physical activity in a year without lockdowns (2019) and comparing it to the corresponding periods in a year with lockdowns (2020) we found that the observed declines in step counts were specifically associated with lockdown implementations rather than being influenced by temperature and humidity conditions.

The study is the first of its kind to investigate the impact of the COVID-19 pandemic on physical activity in Qatar during and after quarantine using objective pedometer. The use of pedometers provided accurate and objective data relative to physical activity. The study had a large sample size for a country the size of Qatar and a longitudinal design, which allowed for the evaluation of changes in physical activity over time. The use of time series was also a key factor for assessing variation in steps around the main events.

Certain limitations also exist and are important to mention. The study was conducted in a specific geographic region and may not be generalizable to other socioeconomic contexts. The sample consisted of participants who were already enrolled in a health promotion program (SIH) revealing a more motivated and health-conscious population, which may limit the generalizability of the findings to the wider population of Qatar. Additionally, the study did not collect data on other factors that may have influenced physical activity, such as socio-demographic characteristics or access to recreational facilities. Considering the limitations of self reported measures to determine BMI, previous work has indicated that BMI computed from self-reported weight and height is a valid measure in adult men and women (32). Future studies should focus on identifying the specific factors contributing to decreased physical activity levels among the most affected sub-groups during pandemics and lockdowns and developing targeted interventions to promote and facilitate physical activity during and after the pandemic.

## 5 Conclusion

The present study is unique in that it uses pedometer data from the “Step into Health” walking program to objectively measure physical activity levels before, during and after quarantine in Qatar, reporting the first evidence in the Gulf Peninsula region. The results show that step counts steeply fell during lockdown and continued to be lower than pre-pandemic levels even after restrictions were lifted. While several studies showed greater impact of lockdown on physical activity levels of women and obese populations, the present study provides new and different insights. Indeed, we observe a larger impact of the lockdown for men, normal-weight and older groups as compared to women, obese and younger groups. This indicates the need to address specific sex, weight-status and age-related differences in physical activity levels during the COVID-19 pandemic according to the context. Strategies to promote physical activity during the pandemic should be tailored for all individuals to have opportunities to maintain their physical activity levels. Findings of this study can inform future health policies and interventions during crises to focus not only on the more vulnerable groups (e.g., obese and women) but also on those who are active at baseline and potentially more sensitive to such lockdown measures. Additionally, exploring factors contributing to the resilience observed in certain groups may guide the development of effective strategies for maintaining physical activity levels in diverse populations.

## Data availability statement

The raw data supporting the conclusions of this article will be made available by the authors, without undue reservation.

## Ethics statement

The study involved humans and was approved by the Institutional Review Board of Aspire Zone Foundation (E202104021) (Doha, Qatar). The studies were conducted in accordance with the local legislation and institutional requirements. The ethics committee/institutional review board waived the requirement of written informed consent for participation from the participants due to study involving the analysis of pre-existing data and the data being anonymized. Written informed consent was not obtained from the individual(s) for the publication of any potentially identifiable images or data included in this article because Patient consent was waived due to study involving the analysis of pre-existing data and the data being anonymized.

## Author contributions

AA-M: Conceptualization, Funding acquisition, Project administration, Resources, Supervision, Writing – original draft, Writing – review & editing. AF: Data curation, Formal analysis, Investigation, Methodology, Resources, Software, Validation, Visualization, Writing – review & editing. AS: Investigation, Resources, Validation, Writing – review & editing. AG: Investigation, Resources, Validation, Writing – review & editing. SA-H: Investigation, Methodology, Resources, Writing – review & editing. LM: Investigation, Methodology, Resources, Validation, Visualization, Writing – original draft. SS: Conceptualization, Investigation, Methodology, Resources, Validation, Writing – review & editing. MC: Conceptualization, Investigation, Methodology, Project administration, Resources, Supervision, Validation, Visualization, Writing – review & editing.
