# Supplementary material for: The effects of the first wave of COVID-19 restrictions on physical activity: a longitudinal study from “step into health” program in Qatar
Source: Front Public Health. 2024 Mar 6;12:1333546. doi: 10.3389/fpubh.2024.1333546 (PMC10951068; doi:10.3389/fpubh.2024.1333546)
Supplement: Supplementary file 1 [file Table_1.DOCX]

**Table.** Timeline of COVID-19 and nationwide restrictions.

| Time point | Date | Event | Details |
| --- | --- | --- | --- |
| 1 | 29 Feb 2020 | First confirmed case of COVID-19 | Qatar’s first ever Covid-19 case was recorded in a 36-year-old Qatari male returning from Iran. |
| 2 | 12 Mar 2020 | Qatar begins to implement lockdown | March 13, 2020– Cinemas, theatres, children’s play areas, gyms and wedding venues, including those in hotels, were closed. |
| 3 | 15-Jun 2020 | Gradual lifting of COVID-19 restrictions: Phase 1 | Limited mosque opening with precautions.  40% capacity for private healthcare facilities.  Partial mall shop openings.  Restricted outdoor sports in parks.  20% workplace capacity with health precautions. |
| 4 | 01-Jul-2020 | Gradual lifting of COVID-19 restrictions: Phase 2 | Small gatherings allowed (≤ 10 people).  Further mosque openings.  Private health clinics open 60%.  Parks, Corniche, and beaches accessible.  Malls open with limits.  50% workplace capacity with health precautions. |
| 5 | 9-Jul-2020 | Gradual lifting of COVID-19 restrictions: Phase 3 | Medium gatherings allowed (≤ 40 people).  Friday prayers in mosques.  Private health clinics open 80%.  Sports training allowed (up to 5 people).  50% capacity for health clubs, gyms, pools, beauty, and barber shops.  Full hours for all malls.  Restricted restaurant openings with gradual capacity increase. |
|  |  |  |  |
