# Supplementary figures and images for: The effects of the first wave of COVID-19 restrictions on physical activity: a longitudinal study from “step into health” program in Qatar
Source: Front Public Health. 2024 Mar 6;12:1333546. doi: 10.3389/fpubh.2024.1333546 (PMC10951068; doi:10.3389/fpubh.2024.1333546)

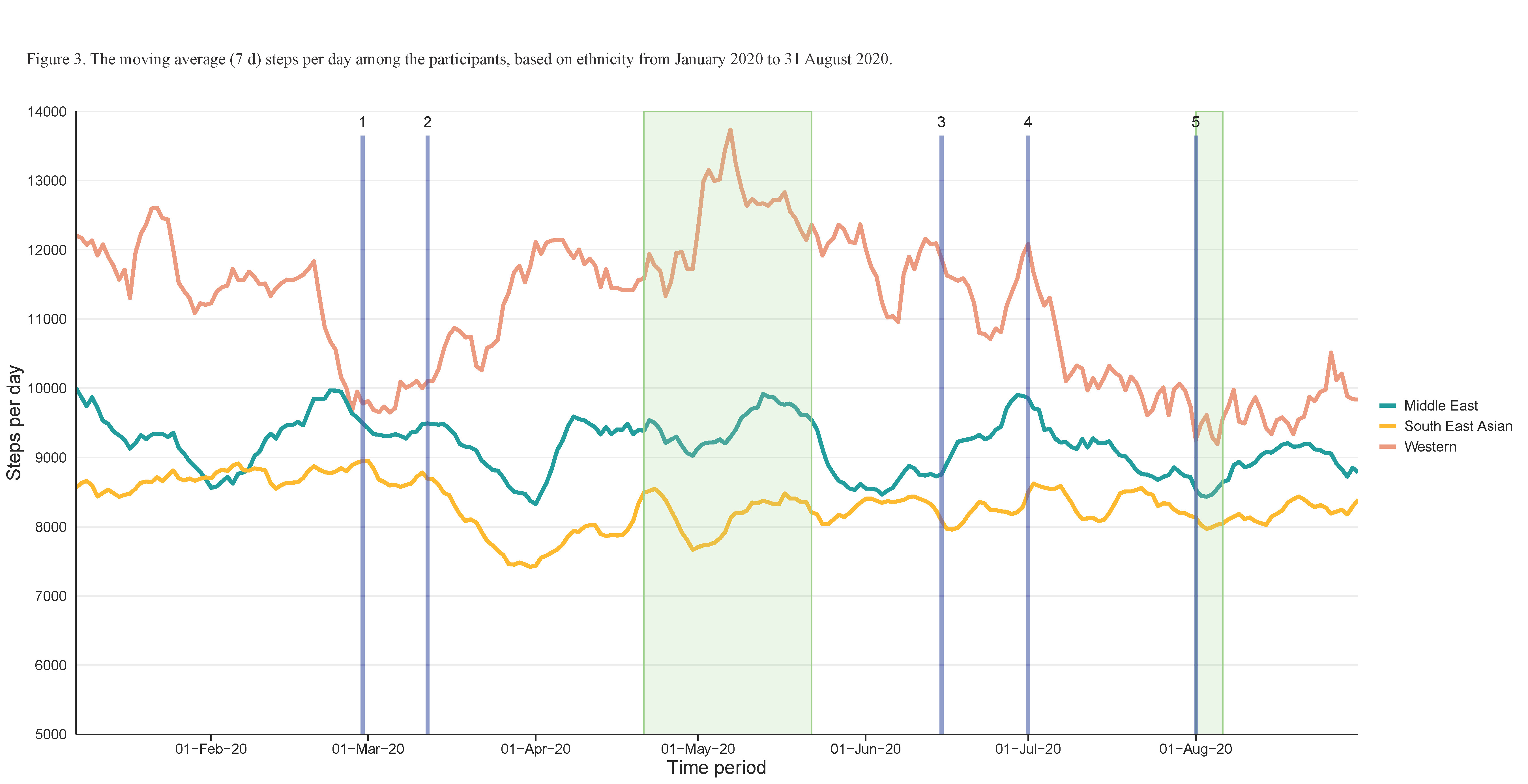

Supplement: Supplementary file 2 [file Image_1.JPEG]
